# Future prospects in radiation oncology from the perspective of innovative radiation biology

**DOI:** 10.1007/s00066-023-02163-w

**Published:** 2023-11-24

**Authors:** Franz Rödel, Udo Gaipl

**Affiliations:** 1 Department of Radiotherapy and Oncology, Goethe University Frankfurt, University Hospital, Frankfurt am Main, Germany; 2 Translational Radiobiology, Department of Radiation Oncology, Universitätsklinikum Erlangen, Friedrich-Alexander-Universität Erlangen-Nürnberg, Erlangen, Germany

Current radiation biology covers a competence to investigate basic and clinical effects of radiation, including radiation protection-relevant issues. By this, growing interest in the complex molecular circuits and in the cellular interactions of different cell types present in cancer and non-malignant tissue has resulted in the emergence of innovative research areas including biological targeting, exploring the impact of the tumor microenvironment (TME) and translational research. This issue joins scientific primary research and review articles to address the landscape of radiobiological research, especially in Europe, covering most of the abovementioned research areas.

While the target structure DNA and mechanisms of DNA damage repair have been a major focus of research, the current horizon is expanding into the extracellular space to modulate the tumor’s micro- and macroenvironment. This paradigm change is illustrated in a review by Frey et al., including a “historical” overview on the evolution of radiation biology spanning “classical” DNA damage sensing and the cellular radiation response, to the interrelationship with the immune system, particularly focusing on the cyclic GMP-AMP synthase cGAS/stimulator of interferon genes (STING) pathway [1]. Further, the review covers the different mechanisms of DNA repair und builds a bridge to current developments including DNA repair inhibitors like poly(ADP-ribose)-polymerase 1 (PARP1) antagonists.

Recent advances in understanding the tumor’s biology in line with a growing number of novel technologies have prompted characterization of patients’ individual malignancies. Although multiple approaches to enhance radiotherapy (RT) or chemoradiation (CRT) effects on tumors using molecular targeting tools have been studied for decades, their translation into clinics is challenging, as reviewed by Viktorsson et al. [2]. Shortcomings are attributed to a lack of suitable biomarkers to select patients for specific treatment approaches, or multifaceted responses with back-up pathways working alongside the targeted ones. Although many strategies have not yet found their way into the clinics, with further progress, RNA- or protein-degrading proteolysis-targeting chimeras (PROTAC)-based targeting and promising developments in the field of nanotechnology will create another way to improve molecular targeted therapy delivery.

A more detailed example of a putative future molecular target is given by Khozooei et al. reporting on the impact of Y‑box-binding protein‑1 (YB-1), a multifunctional protein that tightly governs cell cycle progression, cancer stemness, and DNA damage signaling [3]. Its activity acts in concert with the oncogenic KRAS/YB‑1 cascade, with *KRAS* being among the most commonly mutated oncogenes in human cancers. AKT and p90 ribosomal S6 kinase are downstream of KRAS and are the major kinases that stimulate YB‑1 phosphorylation. Thus, there is a close link between the *KRAS *mutation status and YB‑1 activity.

The group of Scheper et al. [4] provides in vitro data on a tumor-specific radiosensitizing effect of small molecule Ataxia telangiectasia mutated (ATM; AZD0156) and Ataxia telangiectasia and Rad3 related (ATR; VE-822) inhibition in melanoma cells with low toxicity to healthy fibroblasts. Clonogenic survival assays revealed a synergistic radiosensitizing effect and a pronounced G2/M cell cycle arrest by AZD0156, while VE-822 only resulted additive enhancement. ATM inhibition seems here to be superior if combined with RT, with putative consequences for future treatment planning.

Although the cell-autonomous effects of ionizing radiation are well established, there is progressively growing evidence that intercellular communication plays a major role in the outcome of radiation exposure at the tissue level. A combination of RT either with immune stimulants including cytokines or in combination with antibodies that target immune checkpoint inhibitors (ICI), e.g., the programmed cell death protein 1 PD-1/PD-L1 axis is well described. Some of these approaches are currently being tested in clinical trials, enabling huge translational opportunities by examination of the mode of action of RT in combination with immunotherapy.

Despite surgical resection, modern RT, and concomitant temozolomide (TMZ) treatment, glioblastoma multiforme (GBM) patients are still confronted with a very poor prognosis. Thereby, a variety of immune-suppressive mechanisms in GBM represent the major barrier to immunotherapy. Accordingly, knowledge regarding the expression pattern of potential immune checkpoint targets is addressed in the manuscript by Schatz et al. [5]. By in vitro analyses, they indicate that RT modulates the immunogenic phenotype of human GBM cell lines. More particularly, RT but not TMZ results in a significant upregulation of PD-L1, PD-L2, and HVEM, and, interestingly, also stimulates surface expression of additional activating immune checkpoint molecules CD70, CD137L, OX40L, and ICOSL1 that may serve as targets for immune checkpoint blockade in combination with RT.

The aspect of crosstalk between immune checkpoint and DNA damage inhibitors for radiosensitization is covered in more detail in a review by Classen et al. [6]. Tumors with a high tumor mutational burden, PD-L1 expression, or high infiltration of tumor-infiltrating lymphocytes are designated as immunologically “hot” and considered to benefit from immunotherapy. Particularly in cancer cells with deficiencies in DNA repair pathways, homologous recombination, and mismatch repair, increased immunogenicity is reported to correlate with response to ICIs. In consequence, combining ICIs and drugs affecting the DNA damage response (e.g., PARP1, ATM, ATR, and DNA-PKcs inhibitors) with RT may be an attractive option to improve therapies.

Another aspect of the immunomodulatory properties of IR is addressed by a review by Weissmann et al. [7], concentrating on clinically well-established low-dose radiotherapy (LDRT). This option, based on longstanding clinical outcomes, has been in use for decades in patients with inflammatory and/or degenerative diseases. However, the underlying (radio)biological and immunological basics have not yet been completely elucidated. Against this background, the manuscript summarizes the current knowledge on mechanisms associated with clinical effects of LDRT including osteoimmunological properties that have been described in recent years, with macrophages seeming to play a central role.

The most abundant subpopulation of infiltrating immune cells in the TME are tumor-associated macrophages (TAMs). Becherini and colleagues reviewed the current perception on the modulation of TAM activity by RT [8]. Besides its effects on malignant cells, RT can alter TME composition, especially macrophage antitumor (M1) and protumor (M2) polarization. While high-dose irradiation seems to be responsible for angiogenesis and accelerated tumor growth by early M2-TAM infiltration, moderate-dose irradiation stimulates phagocytosis, and a local low-dose treatment may represent a valid option for combination with immunotherapeutic agents. Further, the plasticity of TAMs makes them an attractive target for anticancer therapies and more research should be conducted to explore these potential therapeutic effects.

In addition, the impact of secreted factors after radiation exposure should be followed in more detail for future treatment optimization. Szatmari and coauthors particularly summarize the emerging role of complex extracellular vesicles (EVs) secreted by irradiated cells in the development of radiation-induced effects [9]. EVs are composed of a phospholipid bilayer carrying bioactive molecules including DNA specimens. They can travel certain distances in the body before being taken up by recipient cells and may represent a suitable carrier for future targeted therapeutics.

Epidemiological studies have revealed an increase in cardiac diseases a decade after exposure of the thorax to RT. Hence, Wittmann et al. examined chronic inflammatory effects in primary heart and lung endothelial cells (ECs) of mice after local heart irradiation [10], exemplifying an additional aspect of irradiation exposure, namely normal tissue toxicity. A long-lasting upregulation of prominent inflammatory (HCAM, ICAM‑1, VCAM-1), proliferation (CD105), and lipid (CD36) markers on primary ECs suggests that local irradiation induces chronic inflammation in the microvasculature, resulting in cardiac injury which might be related to an altered lipid metabolism. Consequently, normal tissue effects should be considered when planning future combined-modality treatment.

Another aspect worth further investigation is the use of high linear energy transfer (LET) particle treatment, as the peculiar physical features result in high precision and hence lower toxicity, in line with an increased biological effectiveness. The review article presented by Helm et al. [11] introduces innovative approaches and perspectives, with an emerging role for charged-particle therapy covering FLASH therapy, minibeam irradiation (spatial fractionation), and personalized ultrafractionated stereotactic adaptive RT. Further, a potential for synergies, especially for combination with immunotherapy, has been reported in preclinical studies, as an increased immunogenicity for high-LET particles is anticipated.

The success of allogeneic hematopoietic stem cell transplantation is limited by post-transplant complications such as severe graft-versus-host disease (GVHD) and tumor (re)growth. Finally, the objective of the research article presented by To et al. [12] was to analyze the effect of X‑ray energy levels used for murine total body irradiation prior to semi-allogeneic bone marrow transplantation. Applying a lethal 10-Gy dose with either 6‑MV linear accelerator photons or 160-kV X-rays, their results suggested an equivalence in the capacity to deplete the recipient’s hematopoietic system. Despite this similarity, alloreactive T cell responses varied considerably based on inflammatory cytokine release and is more pronounced after low-energy X-ray treatment. Accordingly, this technical and biological information should be more intensely considered in future research.

In summary, although a complex range of open questions exist, modern radiobiological interdisciplinary and translational research may contribute valuable aspects to planning and optimizing therapeutic concepts and broaden our knowledge about mechanisms that might also be relevant for radiation protection. It’s time for a new way of thinking, with development of innovative solutions to constantly improve personalized therapies and to better understand individual responses.
